# Tumor growth-arrest effect of tetrahydroquinazoline-derivative human topoisomerase II-alpha inhibitor in HPV-negative head and neck squamous cell carcinoma

**DOI:** 10.1038/s41598-024-59592-5

**Published:** 2024-04-21

**Authors:** Patrizia Sarogni, Nicoletta Brindani, Agata Zamborlin, Alessandra Gonnelli, Michele Menicagli, Ana Katrina Mapanao, Federico Munafò, Marco De Vivo, Valerio Voliani

**Affiliations:** 1 Center for Nanotechnology Innovation@ NEST, Istituto Italiano di Tecnologia, Piazza San Silvestro, 12, 56126 Pisa, Italy; 2 Molecular Modeling and Drug Discovery Lab, Istituto Italiano di Tecnologia, via Morego, 30, 16163 Genoa, Italy; 3 NEST – Scuola Normale Superiore, Piazza San Silvestro, 12, 56126 Pisa, Italy; 4 Present Address: Ghent Research Group on Nanomedicines, Department of Pharmaceutics, Ghent University, Ottergemsesteenweg 460, B-9000 Ghent, Belgium; 5 Department of Translational Medicine, University of Pisa, 56126 Pisa, Italy; 6 Fondazione Pisana per la Scienza ONLUS, via Ferruccio Giovannini, 13, 56017 S. Giuliano Terme, Italy; 7 Center for Radiopharmaceutical Sciences, Paul Scherrer Institute (PSI), 5232 Villigen, Switzerland; 8 Department of Pharmacy, School of Medical and Pharmaceutical Sciences, University of Genoa, Viale Cembrano 4, 16148 Genoa, Italy

**Keywords:** Topoisomerase, Head and neck, Alternative models, Tetrahydroquinazoline, Biotechnology, Cancer, Drug discovery, Chemistry, Biomaterials

## Abstract

Oral malignancies continue to have severe morbidity with less than 50% long-term survival despite the advancement in the available therapies. There is a persisting demand for new approaches to establish more efficient strategies for their treatment. In this regard, the human topoisomerase II (topoII) enzyme is a validated chemotherapeutics target, as topoII regulates vital cellular processes such as DNA replication, transcription, recombination, and chromosome segregation in cells. TopoII inhibitors are currently used to treat some neoplasms such as breast and small cells lung carcinomas. Additionally, topoII inhibitors are under investigation for the treatment of other cancer types, including oral cancer. Here, we report the therapeutic effect of a tetrahydroquinazoline derivative (named ARN21934) that preferentially inhibits the alpha isoform of human topoII. The treatment efficacy of ARN21934 has been evaluated in 2D cell cultures, 3D in vitro systems, and in chick chorioallantoic membrane cancer models. Overall, this work paves the way for further preclinical developments of ARN21934 and possibly other topoII alpha inhibitors of this promising chemical class as a new chemotherapeutic approach for the treatment of oral neoplasms.

## Introduction

Head and neck squamous cell carcinomas (HNSCCs) represent a wide class of epithelial neoplasms localized in the oral and nasal cavities, paranasal sinuses, salivary glands, pharynx, and larynx^1,2^. Generally, the strategies for the management of HNSCCs are different between locally advanced and early stage tumors^3^. Indeed, according with the most recent Clinical Practice Guidelines in Oncology (e.g. NCCN), the standard treatments in the early stage of the disease relies on conservative surgery or radiotherapy. Standard options for locally advanced HNSCCs are either surgery plus adjuvant chemo/radiotherapy or primary chemoradiotherapy alone, even if the best treatment regime is selected on a case-by-case analysis^4,5^. In this regard, the drugs of choice for the chemotherapy of HNSCCs are cisplatin-derivatives, even if their sub-optimal efficacy is limited due to the associated severe systemic toxicities (nephrotoxicity, neurotoxicity, ototoxicity, and emesis)^6^. In general, HPV-negative HNSCCs are more aggressive than HPV-positive HNSCC, and locally advanced patients may not respond or even relapse after receiving the gold standard therapy^7^. Indeed, these malignancies are challenging to treat, and up to 40% of patients require modifications of the treatment regimen (i.e., dose reduction, delays, and omissions)^8^. Therefore, there is an urgent need to introduce novel therapies to improve HPV-negative HNSCCs patient outcomes while preserving the structure and function of the involved organs^9–11^.

Topoisomerase include a family of enzymes of different isoforms that participate in DNA replication, transcription and chromatin remodeling^12^. Depending on the differentiation status and topological problems, human cells use a specific type of topoisomerase. Therefore, the replication machinery will be arrested without topoisomerase activity due to supercoiled DNA^13^. In particular, the expression of isoform II alpha is found in actively proliferating cells (phase S and G2/M of the cell-cycle), thus constituting a relevant biomarker for cancers. Indeed, high expression level of TopoIIα indicated poor prognosis for patients with breast cancer and prostate cancer^14,15^. Therefore, interfering with topoisomerase activity may result in an effective therapeutic strategy for cancer as inhibition of both TopoI and TopoII conferred DNA damage and cell death^16^.

In this context, the employment of topoisomerase inhibitors for the chemo-treatment of HNSCCs has been only marginally evaluated for the inhibition of topoI^17^. On the other hand, HNSCCs significantly overexpress topoIIα, pointing out that it can be an interesting target for the treatment of oral neoplasms^18,19^. A recent study has described the development of a new class of tetrahydroquinazoline derivatives as topoII-targeted drugs^20^. These novel compounds differ from those currently used in the clinic as they block the function of topoII without intercalating the DNA, and with no accumulation of the covalent enzyme/DNA cleavage complex, caused by topoII poisons. This mechanism of action may therefore help in avoiding the well-known severe side effects of topoII poisons, which include secondary leukemias in patients^21,22^. In particular, the compound ARN21934 has been shown to prevent DNA relaxation at very low concentrations with ~ 80-fold selectivity for topoIIα over topoIIβ, as the isoform topoIIα is considered the pharmacologically relevant one. ARN21934 also show: (i) broad antiproliferative activity toward cultured human cancer cells, (ii) good drug-like properties and (iii) a favorable in vivo pharmacokinetic profile^20^. Thus, ARN21934 represents a promising compound for further investigations toward the development of more effective and less toxic therapeutic strategies in the treatment of HPV-negative HNSCC.

On these bases, here we present the pharmacological effects of ARN21934 by employing both 2D and 3D in vitro systems, and in vivo chick chorioallantoic membrane (CAM) models of human papillomavirus (HPV)-negative HNSCCs (SCC-25 cell line). CAMs are suitable biomodels for the preclinical assessment of both the antitumor activity and the mechanisms of action of established and emerging chemotherapeutic agents (e.g*.* metal complexes or hybrid nano-architectures) as well as for the evaluation of the neoplasms’ behaviors^23–25^. Indeed, the generation of solid and vascularized neoplasms on the CAM allows for a rapid assessment of the potential tumor-shrinking effect, impairment of vascularization, and/or metastasis modulation of the treatment^26^.

Remarkably, ARN21934 demonstrated a cytotoxic activity in 2D cells at a micromolar concentration as well as a significant growth inhibitory effect in both 3D and in vivo models at the translated dose. The tumor size-reduction effect observed in CAMs occurred with a significant reduction of VEGF-A mRNA, one of the transcription factors that contributes to tumor angiogenesis and growth. We also evaluated other cell cycle regulatory genes and tumor histology to verify the cytotoxic effects of ARN21934. Importantly, this work promotes ARN21934 as a valid lead compound for developing new anticancer therapeutics while further highlighting the suitability of the CAM models in the workflow of oncological research. Overall, this study opens new horizons in the development of novel platinum-free chemotherapeutics strategies for the management of head and neck carcinoma.

## Materials and methods

### Chemistry

ARN21934 was synthesized through the eight steps synthetic route previously described starting from commercially available 1,4-cyclohexanedione monoethylene acetal (cas: 4746-97-8) and *N*,*N*-Dimethyl-*p*-phenylenediamine (cas: 99-98-9)^20^. All of the commercially available reagents and solvents were used as purchased from vendors (Merck) without further purification. ^1^H NMR in DMSO-*d*_6_ analysis of synthesized ARN21934 is consistent with previously reported data (NMR spectra in the supporting information, Fig. S1). Tested compound showed > 98% purity by UPLC/MS analysis (Chromatographic analysis in the supporting information, Fig. S2).

### Cell culture (*2D and 3D*)

Human squamous cell carcinoma SCC-25 was purchased from the American Type Culture Collection (ATCC). SCC-25 cells were maintained in a complete growth medium composed by a 1:1 mixture of Dulbecco’s modified Eagle’s medium and Ham’s F12 medium supplemented with 10% fetal bovine serum (FBS), 4 mM L-glutamine, 1 mM sodium pyruvate, 100 U/mL penicillin, 100 mg/mL streptomycin (Invitrogen) and 400 ng/mL of hydrocortisone. Cells were maintained at 37 °C in a humidified 5% CO_2_ atmosphere. For 3D structures, we used the optimized protocol from Santi et al.^27^. Briefly, cells were harvested and centrifuged for 5 min at 1200 rpm and then resuspended in fresh medium and counted. The suspension was adjusted to a final concentration of 1 × 10^6^ cells/mL. Afterwards, 10 μL of cells suspension were placed on the lid of a 100-mm cell culture dish that was flipped into the dish containing 10 mL of PBS. Cells drops were left to settle in the incubator until they formed a sheet and after 3 days were then transferred to a 100 mm suspension culture dish. Finally, cells aggregates were placed inside a CO_2_ incubator with an orbital shaker (70 rpm) for 24 h to induce the formation of the proper spherical shape.

### Viability assay on 2D cell culture

The cytotoxicity of the tetrahydroquinazoline derivative ARN21934 and cisplatin was evaluated by using a tetrazolium salt, 2-(2-methoxy-4-nitrophenyl)-3-(4-nitrophenyl)-5-(2,4-disulfophenyl)-2H tetrazolium, and monosodium salt (WST-8) assay. SCC25 cells were seeded in 96-well plate and cultured for 24 h to let the cells adhere. The cells were then incubated in the presence of ARN21934 or cisplatin at increasing concentration in medium (100 μL) for 2 h at 37 °C. After incubation, the drug was removed and cells were washed twice with PBS and kept in fresh medium. For each experimental time point cells were incubated with 10% WST-8 reagent (10 µL) and 10% serum-containing medium (90 µL) for 2 h. Absorbance (450 nm) was measured using a microplate reader (Glomax Discovery, Promega, Madison, WI, USA). The percentage of cells viability was determined by comparing the drug-treated cells with the untreated cells (control, 100% viability). Data represent the average of 2 independent experiments each with 3 experimental replicates. Error bars represent the SD from two independent experiments.

### Viability assay on 3D cell models

Viability of 3D spheroids was evaluated using the CellTiter-Glo® 3D Cell Viability Assay (Promega, Milan, Italy). Briefly, 3D spheroids were divided by type of treatment and incubated with increasing concentration of ARN21934 (20X with respect to the 2D model) for 2 h at 37 °C. Treated or untreated spheroids for each time point were transferred separately to a white 96-well plate for luminescence measurements with 100 µL of medium. Then, 100 µL of CellTiter-Glo® 3D reagent was added to each well, the plate was shaken for 5 min and the luminescence signal was recorded after 25 min of incubation with a microplate reader (Glomax Discovery, Promega, Madison, WI, USA). Cell viability was determined with respect to the untreated spheroids. Data and error bars represent the average and SD of three independent experiments respectively.

### Proliferation assay

The inhibitory effect of ARN21934 was evaluated through the cell counting using the trypan blue method in a time-course experiment. The SCC-25 cells were trypsinized and counted such that 2 × 10^5^ cells were seeded into p35 mm dishes (0 h). The following day, 0.8 µM solution of ARN21934 was added to the treated group for 2 h at 37 °C. The inhibitory effects were evaluated after 24, 48 and 72 h after exposure with the drug.

### SCC-25 tumor-grafted chorioallantoic membrane (CAM) assay and tumor monitoring

The optimized tumor grafting procedure with SCC25 cells on CAM model has been previously reported and discussed in Sarogni et al.^2^. Briefly, fertilized red Leghorn chicken eggs were immediately stored at 4 °C upon delivery. Before starting the incubation, the eggs were cleaned with deionized water and then placed in trays inserted in a fan-assisted incubator (FIEM MG 140/200) set at 37.5 °C with ∼ 47% humidity. The Embryonic Day of Development (EDD) 0 was assigned to the day of the start of incubation. On EDD3, eggs were punctured with a small hole on the top, sealed with an adhesive tape and placed back in the incubator in upright position. On EDD6 the small hole was further cut into a small window of about 1 cm^2^ to facilitate cells positioning. The implanted cells were suspended in 1:1 mixture of serum-free medium and Matrigel (Corning 354,234). Each egg was then grafted with 2 × 10^6^ cells suspended in 25 μL of grafting mixture and returned in the incubator. Four days post-grafting (EDD10), tumor-bearing embryos were randomized and divided into three groups of study: serum-free medium-treated eggs (control) and ARN21934-treated eggs, and cisplatin-treated eggs with at least *n* = 10 eggs per condition. Each tumor xenograft of the ARN21934-treated group received 3.5 μg of the drug compared to 6 μg for cisplatin-treated group. The tumors of both groups were monitored and photographed on EDD12 and EDD14 using a portable digital microscope (Dino-Lite AM7915MZT) and tumor dimensions were determined using a complementary software (DinoCapture 2.0). Tumors harvesting was executed after placing the eggs at 4° C at least 2 h to induce hypothermia and restrict chicken embryos movements. The collected tumors were stored at -80 °C or used immediately for further analyses. The data and statistical analyses were analyzed using GraphPad Prism 9. All the experiments have been performed in compliance with the current European and Italian laws (directive 2010/63/UE, and D. Lgs. 26 March 4^th^, 2014) on the protection of animals used for scientific purposes and do not require ethical approval as chick embryo is not considered as living animal until day 17^28^.

### Quantitative real-time PCR

The harvested tumors were carefully minced into pieces using a plastic pestle, and the total RNA was extracted using Nucleospin RNA plus Kit (740984.50 MACHEREY–NAGEL) following the manufacturer’s instructions. The extracted RNA was quantified using a nanodrop instrument (UV5NANO Mettler-Toledo) and the quality was then verified through agarose gel electrophoresis. The RNA was stored at − 80 °C or immediately used. Five hundred nanograms of RNA was reverse transcribed for cDNA synthesis using iScript cDNA Synthesis Kit (Biorad 1708891). Reverse transcription was followed by quantitative Real-Time PCR using iTaq™ Universal SYBR® Green Supermix (Biorad 1725121). Thus, 500 ng of the total cDNA was diluted 1:10 to have a final concentration of 2.5 ng/μL and 1 μL of the diluted cDNA was used for the preparation of RT-PCR samples and amplification of each target gene. PCR mix was prepared such that each sample had a final volume of 10 μL. Glyceraldehyde 3-phosphate dehydrogenase (GAPDH) was used as housekeeping gene. Primers used for gene analysis were the following: PCNA-Forward: 5′-CGGATACCTTGGCGCTAGTA-3′; Reverse: 5′-CACTCCGTCTTTTGCACAGG-3′; Caspase-3-Forward: 5′-CAAACTTTTTCAGAGGGGATCG-3′, Reverse: 5′-GCATACTGTTTCAGCATGGCAC-3′; VEGF-A-Forward: 5′-GGGCAGAATCATCACGAAGT-30, Reverse: 5′-TGGTGATGTTGGACTCCTCA-3′; GAPDH-Forward: 5′-AGAAGGCTGGGGCTCATTT-3′, Reverse: 5′-AGTCTTCTGGGTGGCAGTGAT-3′. All samples were analyzed in duplicate. The amplification curves were visualized using SYBR Green Analysis on Applied Biosystems Instrument (7300). The recommended thermal cycling program for amplification is as follows: 95 °C for 10 min and 40 cycles at 95 °C for 15 s, 60 °C for 30 s and 72 °C for 30 s. Relative gene expression levels were calculated using 2 − ΔΔCt method^29^.

### Hematoxylin and eosin (H&E) staining

Tissues were fixed in 10% buffered formalin for at least 48 h. Sections of 4 μm thickness were obtained from paraffin blocks using a microtome (Leica RM2255 Germany). Samples were dewaxed in xylene for 5 min, dehydrated with ethanol solutions (5 min each), and then stained with hematoxylin for 5 min (Mayer hematoxylin, Diapath C0303), and eosin for 1 min (Eosin G o Y alcoholic 0.5% Diapath C0353) and re-immersed in alcohol and xylene. Slides were mounted using a synthetic mounting media (Thermoscientific, LAMPB/DPX).

### Immunohistochemitry (IHC)

Deparaffinized samples were treated for 10 min with hydrogen peroxide solution (3%) to stop peroxidase activity. Thereafter, samples immersed in EDTA-based buffer (pH 8.0) (Leica Biosystems RE7116-CE) were treated in a microwave oven (10 min, 480 W) for antigen retrieval. Nonspecific staining was prevented with blocking peptide (Abcam HRP/DAB, detection kit, ab 64,261). The primary antibodies for Ki67 (rabbit monoclonal Invitrogen MA5-14,520, diluted 1:100) and cleaved Caspase 3 (rabbit polyclonal Cell Signaling Technology, 9661S; diluted 1:500) were applied and left overnight at 4 °C. The detection was performed using streptavidin–biotin technique (Abcam HRP/DAB, detection kit, ab 64,261). Finally, the chromogen (diaminobenzidine) was used for IHC development and Mayer’s hematoxylin for the counterstaining. To quantitatively determine the protein expression, IHC images were processed with a specific algorithm in Aperio ImageScope software (Positive Pixel Count). The signal intensity was determined by averaging three different area of the slide section. Therefore, the percentage of Positivity was automatically determined and included the sum of three different signal intensity: weak, moderate, and strong.

## Results and discussion

### In vitro efficacy evaluation of ARN21934

**Figure 1:**
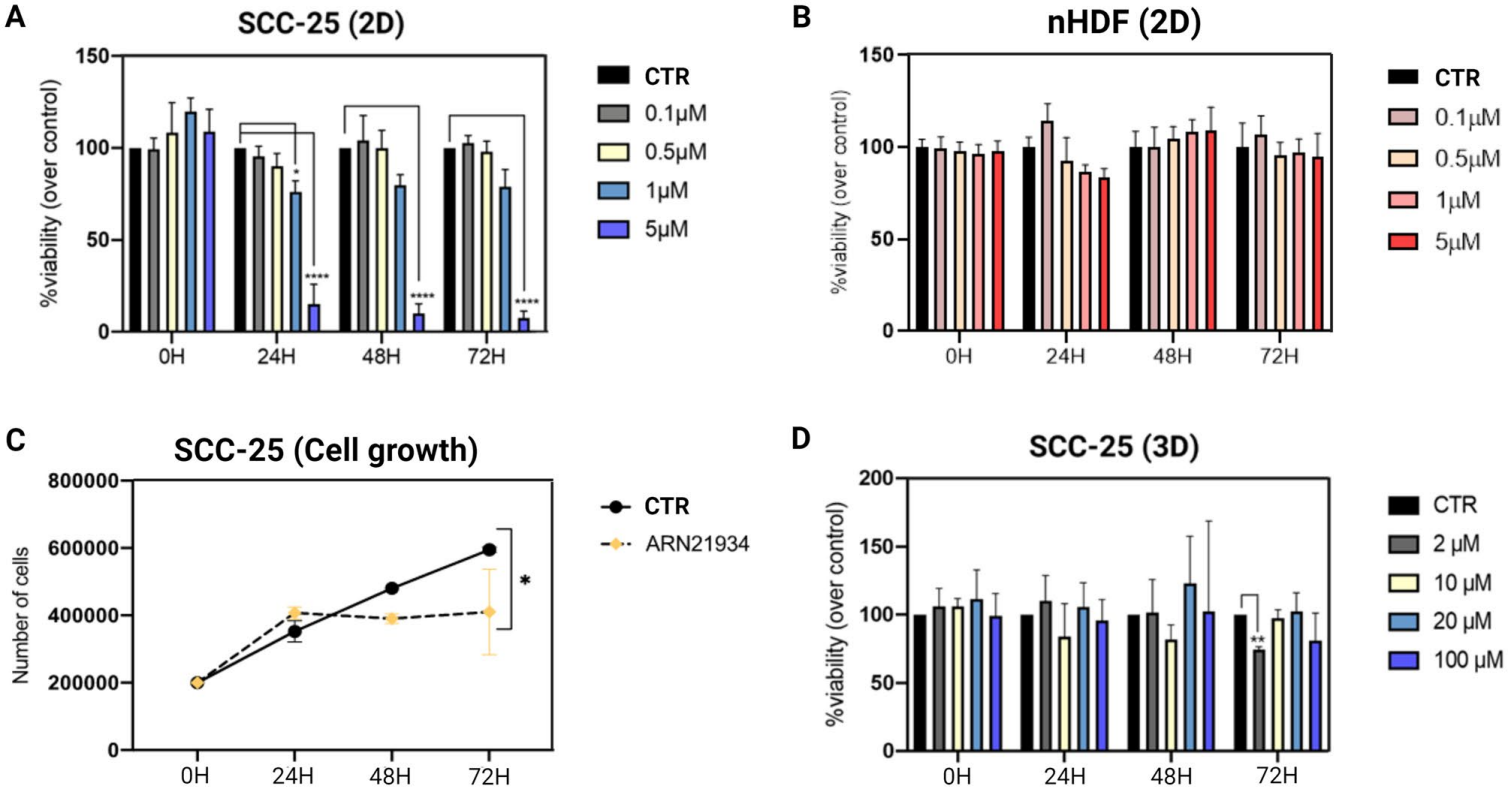
Antitumor effect of ARN21934 tetrahydroquinazoline derivative on SCC-25 cells. (**A**) Cell viability was measured at 24 h, 48 h and 72 h after treatment with ARN21934 for 2 h at 37 °C. Data were normalized over the viability of control cells treated with medium. Data are reported as mean + SD of two biological specimen, each with three experimental replicates. Statistical differences were calculated with two-way ANOVA (Tukey’s multiple comparison test; ****p < 0.0001. (**B**) ARN21934 compound was tested on Human Dermal Fibroblast (nHDF) cells. Cell viability was measured at 24 h, 48 h and 72 h after treatment for 2 h at 37 °C. Data were normalized over the viability of control cells treated with medium. Data are reported as mean ± SD calculated as propagation error of one biological replicate. (**C**) Cells growth inhibition observed within 72 h after treatment with a dose of ARN21934 below the IC50 value (0.8 μM) with respect to the control. Significant proliferation inhibition of ARN21934-treated cells was observed at 72 h with respect to the control. Statistical differences were calculated though the Sidàk’s multiple comparisons test, *p < 0.01. (**D**) Spheroids were treated with 20-fold higher doses than 2D cells for 2 h at 37 °C. Data were normalized over the viability of untreated spheroids and reported as mean + SD of three biological replicates. Statistical differences were calculated with Student’s t-test, **p < 0.01.

The effects of ARN21934 on cancer cells was evaluated in vitro by using an HPV-negative head and neck cancer cell line (SCC-25). A significant viability reduction within 24 h has been observed in the 2D system after two hours incubation with 5 μM of the compound (IC_50_: 1.93 μM) (Fig. 1A). This treatment regime was derived from the clinically approved etoposide dose to treat small cells lung cancer patients^30^. Interestingly, the cytotoxic efficacy was still observed after 48 and 72 h with a similar IC_50_ value (IC_50_^48h^: 1.9 μM and IC_50_^72h^: 1.74 μM). These findings suggest a cytotoxic effect of ARN21934 at lower doses compared to Cisplatin^10^. Noticeably, the short drug exposure timeframe ensures an adequate preclinical evaluation of its antitumor effect, as the continuous and/or prolonged incubation limits the recovery of the cells and might interferes with the interpretation of the data^31^. In comparison, normal human dermal fibroblast cells (nHDF) were employed as a control cell line in the same setup as one of the most representative constituents of organs^32^. The toxicity observed in SCC-25 cells was not found in nHDF cells, confirming the safety profile of ARN21934 (Fig. 1B). Moreover, a 2D proliferation assay was performed to evaluate the inhibitory effect of ARN21934 when used at a non-cytotoxic dose (0.8 μM, below the IC_50_ value). A significant inhibition of the cell growth was observed up to 72 h in ARN21934 treated samples compared to the control (Fig. 1C).

Next, we employed multicellular tumor spheroids (MCTS) to better represent some physiological behaviors of the neoplasm, among which are oxygen and nutrients gradients, and the extracellular matrix (ECM)^33^. Standard production protocols for HPV-negative squamous cells carcinoma MCTS establishment and drug assessments have been employed^10,25,27^. It should be noted that cell–cell and cell-ECM interactions, which characterize the complex permeability barriers of MCTS and tumors in vivo*,* may delay the drug penetration and response to treatments, triggering drug resistance mechanisms^34^. For this reason, the concentration of ARN21934 for the treatment of MCTS was increased × 20 compared to 2D cell cultures. Despite the dose escalation, the viability of treated MCTS was relatively comparable to the controls for each experimental time point except for the condition 2 μM at 72 h (Fig. 1D). The contrasting effects observed between 2 and 3D models suggests that the complexity of the system may influence the treatment response, and highlights the different tolerances to the drugs of 3D systems, which may properly recapitulate the in vivo sensitivity to the treatments. In this scenario, we assumed that ARN21934, which has a cytotoxic effect on 2D cells, may inhibit proliferation of cells in MCTS.

### CAM assay

**Figure 2:**
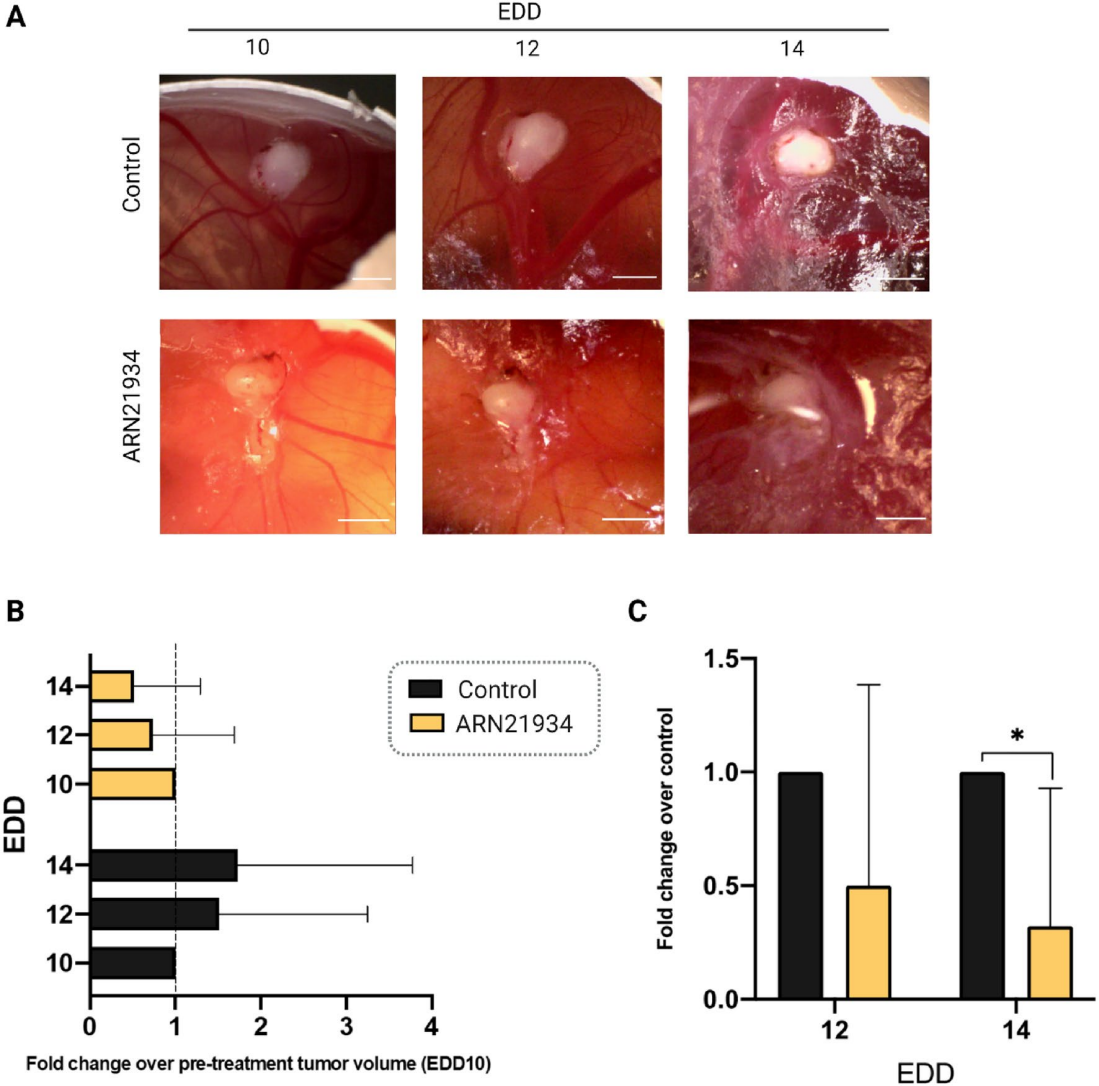
Effect of ARN21934 on tumor size in in vivo chick chorioallantoic membrane cancer model. (**A**) Representative images of tumors grown on CAM. Tumors were monitored from EDD10 to EDD14. Scale bar: 2 mm (**B**) Tumor volume was calculated as fold change over the pre-treatment tumor size at Day 10 for both groups. Data are reported as mean + SD in which *n* = 11 and *n* = 17 for the control and ARN21934 group, respectively. No statistical differences were found among the EDDs for both groups. The dashed line refers to the fold change and it is set equal to 1. (**C**) Tumor volume shrinking effect was evaluated as compared to the control. The average tumors volume of ARN21934-treated group was divided for the average tumor volume of the control for both EDD12 and 14. Error bars are propagation of the previously calculated SD. Statistical differences were calculated with two-way ANOVA (Tukey’s multiple comparison test, *p < 0.05).

The antitumor activity of ARN21934 was further evaluated by employing our optimized alternative in vivo model for head and neck carcinoma (CAMs)^2^. After randomization and distribution of tumor-bearing embryos in the two groups of treatment, 3.5 μg of ARN21934, which corresponds to 9.7 nmol, were topically administered to each tumor xenograft. The treatment for the in vivo experiment was derived from the etoposide dosage generally administered intravenously (50–100 mg/m^2^) for several malignancies and considering the conversion factor between the human average body weight and surface area^30,35^. Then, the clinically applied dosage of the drug has been employed in the in vivo treatments by considering the weight of the chicken embryo at EDD10 (2.3 g) without applying the interspecies allometric scaling^23^. At EDD10, the surface dimensions of the tumors were estimated to calculate the tumor volume, and employed to determine the fold change value (Fig. S3A)^23^. The individual size distribution of tumors treated with the inhibitor revealed a drastic shrinkage of most of them (Fig. S3B, *right*). Therefore, a progressive volume reduction of the tumors treated with ARN21934 was observed (Fig. 2A and B), and resulted comparable to cisplatin-treated samples (Fig. S4). The data obtained from ARN21934 treated samples normalized over the controls confirmed the significant tumor treatment response (Fig. 2C). This result is of special interest for the next clinical applications such as the establishment of single or combined therapeutic approach also with inhibitors of topoI, which already demonstrated significant activity in the treatment of metastatic or recurrent squamous carcinoma of the head and neck^17^.

**Figure 3:**
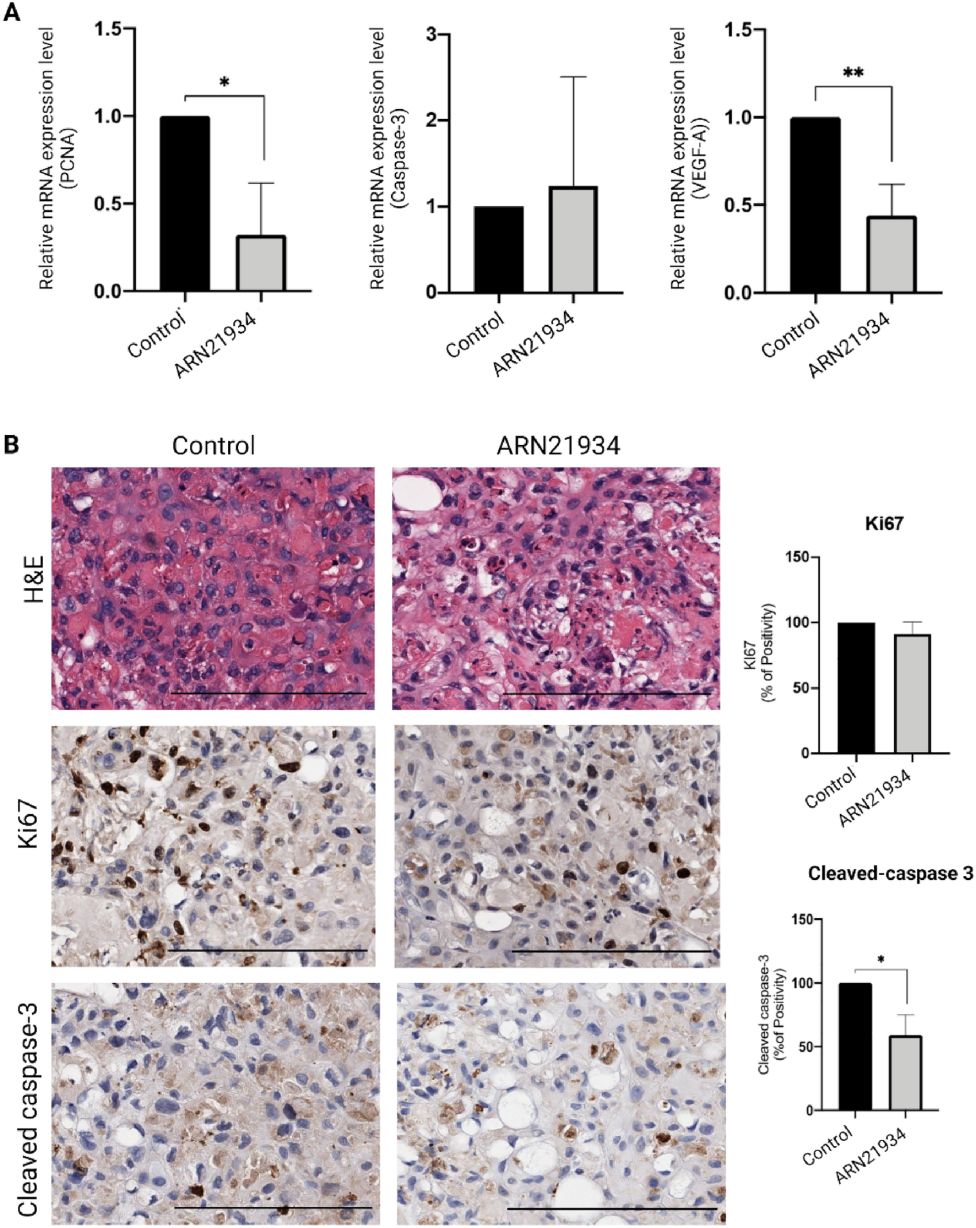
Evaluation of cell-cycle mechanism. (**A**) Comparison of PCNA (*left*), Caspase-3 (*middle*) and VEGF-A (*right*) between the two groups of study. Data is reported as mean + SD of three biological replicates. Statistical differences were calculated through the Student’s t-test, *p < 0.05, **p < 0.005. (**B**) Proliferation and apoptosis were further examined through IHC with anti-Ki67 (*middle*) and anti-Cleaved caspase 3 (*bottom*) antibodies for both groups. Scale bar: 200 μm. The brown signal in the IHC images was quantified with the Positive Pixel Count Algorithm in the Aperio ImageScope Software. The percentage of Positivity was then automatically generated and averaged among three different area of the tissue section. Statistical difference was calculated through the student’s t-test (unpaired, Welch’s correction, *p < 0.05) H&E staining (*left*) denoted undamaged tissue for the ARN21934-treated group.

On EDD14 the tumors were collected and processed for further biomolecular investigations in order to shed light on some mechanism of action of ARN21934. Indeed, while drug-induced cytotoxicity promotes a programmed cell-death mechanism, i.e*.* apoptosis, the anti-proliferative action of certain drugs may induce senescence and inhibition of genes involved in cell proliferation, resulting in tumor growth arrest effect^36,37^. In this regard, a significant reduction of PCNA mRNA expression levels in ARN21934-treated tumors denoted an inhibitory effect on the DNA synthesis, causing a proliferation arrest (Fig. 3A, *left*)^38^. This finding is consistent with the anti-proliferative activity of other clinically approved topoisomerase inhibitors for the treatment of colorectal, gastric and small cell lung carcinoma, such as etoposide VP-16, Adriamycin, and Irinotecan^39^. On the other hand, a very low variation of caspase-3 mRNA expression was observed, implying that further investigations regarding the cleaved form of the protein implicated in the apoptotic mechanism are required (Fig. 3A, *middle*). Among other factors involved in cancer progression, angiogenesis plays a key role in supplying nutrients and oxygen by promoting tumor growth^40^. On this regard, it has been demonstrated that VEGF-A isoforms contribute to oral cancer progression and tumor differentiation^41^. Hence, the significative reduction of VEGF-A mRNA expression after the treatment with ARN21934 may suggest a potential activity of the topoII inhibitor in controlling the tumor-induced angiogenesis mechanism (Fig. 3A, *right*), even if more evidence are required. In general, conflicting evidence on the effect of topoI inhibitors derivatives are reported on angiogenesis. For example, Xu et al.^42^ demonstrated the anti-angiogenic properties of an indenoisoquinoline derivative with high inhibitory activity of topoI, which significantly reduced blood vessels branching on the surface of the CAM. On the other hand, Ethoxidine (identified as a potent topoI inhibitor) increases VEGF expression in human endothelial cells, suggesting a contrasting effect in which the anti-proliferative properties are associated to the generation of a vascular network^43^.

Proliferation and apoptotic behaviors of the tumors were further assessed through immunohistochemistry (IHC) by respectively employing antibodies for Ki67 and the cleaved form of caspase-3. The quantitative analysis revealed a significant reduction of the cleaved caspase-3 protein, suggesting that the tumor shrinking effect might be due to a proliferation arrest more than the activation of apoptotic mechanisms (Fig. 3B). On the other hand, ARN21934 did not show relevant effects on the expression of Ki67, suggesting that further biological evaluations would be required to unveil the relationship between the compound and the cell cycle (Fig. 3B). However, the different PCNA mRNA expression and Ki67 protein levels might be attributed to their different role in this cell-cycle regulation process. Indeed, while Ki67 is preferentially expressed during the late G1, S, G2, M of the cell-cycle, PCNA is elevated during the G1/S phase as it is an ancillary protein for DNA polymerase alpha^44^. Moreover, PCNA protein participates in DNA synthesis and is a component of DNA repair mechanism^45^. Despite the functional differences of these two proteins, they are both commonly used to investigate the cell mitotic index and tumor grading^46^.

As expected, Hematoxylin and Eosin (H&E) staining of the tumor tissues did not reveal damages following the application of ARN21934 with respect to the control, further corroborating the absence of a toxic effect of the compound on cells morphology and their organization (Fig. 3B).

Interestingly, this finding strongly differs from the effects associated with the treatment of CAMs with cisplatin (the standard of care for HNSCCs) that highlighted a loss of organization of the tumor tissue and hardly identifiable cells^23^. In this regard, the biocompatibility of ARN21934 has been further confirmed by the embryo viability (94%) versus cisplatin-treated CAMs (80%) (Fig. S5). Indeed, only one tumor-bearing embryo died two days after the administration of ARN21934. This result is of particular relevance by considering that the overall antitumor activity of ARN21934 on CAMs is comparable to cisplatin^23^.

## Conclusion

In summary, we have evaluated the therapeutic effect of the topoIIα inhibitor ARN21934 for the treatment of HPV-negative head and neck squamous cell carcinoma. The antitumor activity of this lead compound has been assessed in both in vitro and in vivo models, denoting a significant and concentration-dependent effect. Notably, the inhibitory effect on the chick chorioallantoic membrane (CAM) cancer models was confirmed by the reduction of the expression of both PCNA mRNA and capsase-3 protein active form. Since topoII inhibitors have also been shown interesting clinical application when used in combination with other drugs, such as HDAC inhibitors and microtubule stabilizers, our results suggest that ARN21934 may also be considered for further evaluations in combined chemo-treatments of HNSCCs^47^. The combined chemotherapy might prevent the replication of cancer cells that survived the effect of established chemotherapeutic agents. On a broader basis, ARN21934 represents a promising starting point for new chemo-treatment strategy for oral neoplasms that may reduce the employment of platinum-based drugs.

## Limitation of the study

This study provides a comprehensive overview of the antitumor effects of a new class of topoisomerase inhibitor compound in 2D, 3D, and alternative in vivo models of HNSCCs. Nevertheless, further advanced biological investigations may shed light on the molecular mechanisms and signaling pathways involved (i.e., DNA damage repair, ROS production, metastasis regulation, EGFR/MAPK). The CAM model is a suitable and reliable alternative in vivo system for oncology investigations that agrees with the 3R principles (Reduction, Refinement, Replacement) on the use of animals in preclinical research. Obviously, some drawbacks have to be considered, such as the short experimental window for the screening of new drugs as well as the technical difficulties in administering consecutive therapies. The absence of a fully developed immune system does not allow a complete representation of tumor-immune cells interactions, limiting investigations of the inflammatory responses. For angiogenesis-related studies, it may result quite difficult to distinguish between the formation of newly formed vessels and the pre-existing ones by considering the limited availability of anti-chicken antibodies. Moreover, their employment in some research fields may require the establishment of new ad hoc procedures, such as in radiotherapy investigations^48^. However, CAMs represents a powerful tool in preclinical and translational cancer research to evaluate and compare both established and emergent treatment approaches for the next cancer management.

### Supplementary Information

Supplementary Figures.

## Data Availability

All data generated and analyzed during this study are included in this published article and its supplementary information files. The raw data are available from the corresponding author on reasonable request.
