# Supplementary material for: Tumor growth-arrest effect of tetrahydroquinazoline-derivative human topoisomerase II-alpha inhibitor in HPV-negative head and neck squamous cell carcinoma
Source: Sci Rep. 2024 Apr 21;14:9150. doi: 10.1038/s41598-024-59592-5 (PMC11033276; doi:10.1038/s41598-024-59592-5)
Supplement: Supplementary file 1 — Supplementary Figures. [file 41598_2024_59592_MOESM1_ESM.docx]

**Supplementary Information**

**Tumor Growth-Arrest Effect of Tetrahydroquinazoline-derivative Human Topoisomerase II-alpha Inhibitor in HPV-Negative Head and Neck Squamous Cell Carcinoma**

Patrizia Sarogni,^1^ Nicoletta Brindani,^2^ Agata Zamborlin,^1,3,4^ Alessandra Gonnelli,^1,5^ Michele Menicagli,^6^ Ana Katrina Mapanao,^7^ Federico Munafò,^2^ Marco de Vivo,^2,*^ and Valerio Voliani,^1,8,*^

^1^ Center for Nanotechnology Innovation@ NEST, Istituto Italiano di Tecnologia, Piazza San Silvestro, 12 - Pisa (56126), Italy

^2^ Molecular Modeling & Drug Discovery Lab, Istituto Italiano di Tecnologia, Via Morego, 30 – Genoa (16163), Italy

^3^ NEST – Scuola Normale Superiore, Piazza San Silvestro, 12 - Pisa (56126), Italy

^4^ Current address: Ghent Research Group on Nanomedicines, Department of Pharmaceutics, Ghent University, Ottergemsesteenweg 460, B-9000 Ghent, Belgium.

^5^ Department of Translational Medicine, University of Pisa - Pisa (56126), Italy

^6^ Fondazione Pisana per la Scienza ONLUS, via Ferruccio Giovannini, 13 - S. Giuliano Terme (56017), Italy

^7^ Center for Radiopharmaceutical Sciences, Paul Scherrer Institute, Villigen-PSI (5232), Switzerland

^8^ Department of Pharmacy, University of Genoa, Viale Cembrano, 4 – Genoa (16148), Italy

*Corresponding Author: [marco.devivo@iit.it](mailto:marco.devivo@iit.it), [valerio.voliani@unige.it](mailto:valerio.voliani@unige.it)

**Figure S1**

**
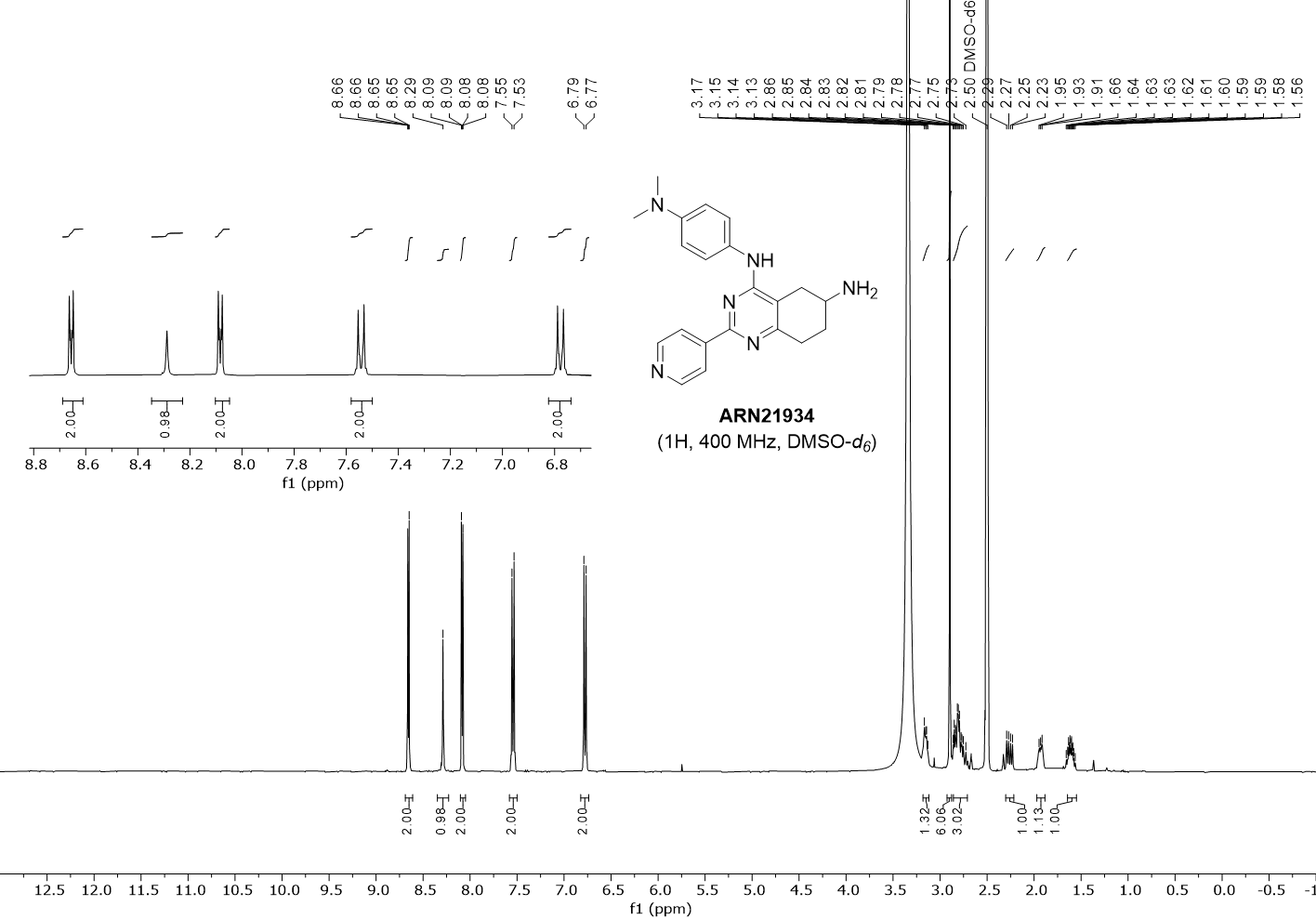
**

**Fig. S1.** NMR spectra of ARN21934

**Figure S2**

**Figure S2.** Chromatographic analysis of ARN21934. After its synthesis, ARN21934 underwent UPLC-UV-MS quality control analysis using reversed phase chromatography. Analyses were conducted on a Waters Acquity UPLC/MS system (Waters Inc. Milford, MA) consisting of a single quadrupole detector (SQD) mass spectrometer equipped with an electrospray ionization interface and a photodiode array detector (PDA). The scan range was set to 110–650 m/z for both polarities (ESI+ and ESI−). The PDA wavelength range was 210–400 nm (bottom image), and the UV purity was determined at the specific wavelength of 215 nm (upper image). The analyses were performed on an Acquity UPLC BEH C18 column (100x2.1mmID, particle size 1.7µm) with a VanGuard BEH C18 pre-column (5x2.1mmID, particle size 1.7µm) using 10 mM NH_4_OAc in H2O at pH 5 adjusted with acetic acid and 10 mM NH_4_OAc in MeCN-H2O (95:5) at pH 5 (B) as mobile phase. After an initial hold for 0.2 min at 10% B, a linear gradient was applied to 90% B in 6 min, then from 90 to 100% B in 0.1 min, followed by a hold at 100% for 0.4 min. A 10 mM stock solution in dry DMSO was prepared for each test compound, and further diluted 20× in MeCN–H_2_O (1:1) prior to analysis.

Rt ARN21934 = 2.43 min, MS (ESI) m/z: 361.3 [M + H]+.

**Figure S3**

**
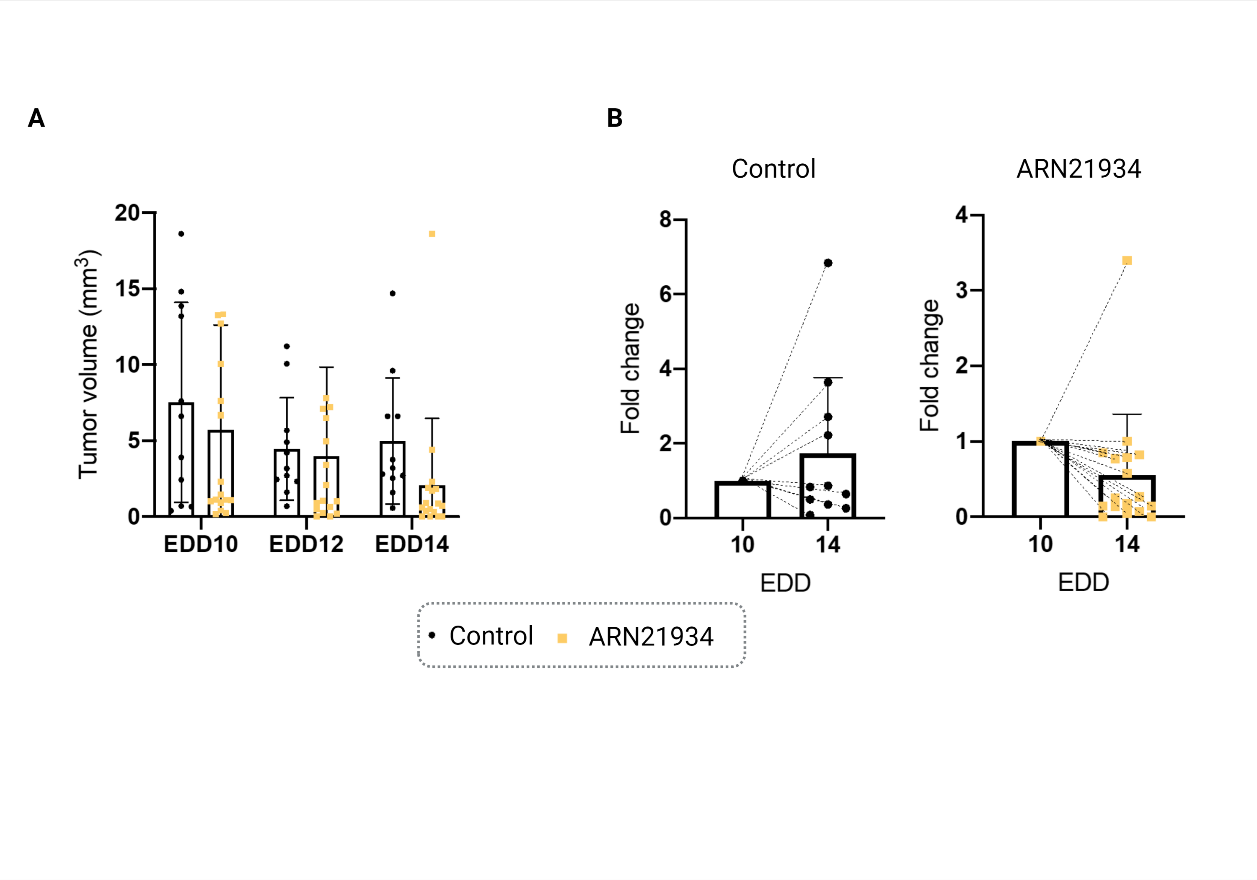
**

**Figure S3.** Tumor volume distribution on CAM model. (**A**) Tumors dimensions were evaluated until EDD14 and estimated through the superficial measurement of width and length. Pictures were taken before the treatment at EDD10. Due to the heterogeneous size distribution between the two groups, the effect on tumor volume was calculated through the fold change value. No statistical differences were found among the groups. (**B**) Individual tumor behavior after normalization over the pre-treatment tumor volume at day 10 (fold change). Data are reported as mean + SD.

**Figure S4**

**Figure S4.** Tumor volume fold change of control and cisplatin-treated tumors over the EDDs. Data are reported as mean ± SD of three independent experiments, including 10 and 20 eggs in total for control and cisplatin group, respectively. No statistical differences were noted among the two groups.

**Figure S5**

**Figure S5.** Viability of tumor-bearing embryos treated with serum-free medium (control), cisplatin (6μg) and ARN21934 (3.5μg).
